# Differences in subthalamic oscillatory activity in the two hemispheres associated with severity of Parkinson’s disease

**DOI:** 10.3389/fnagi.2023.1185348

**Published:** 2023-08-28

**Authors:** Xuemin Zhao, Ping Zhuang, Mark Hallett, Yuqing Zhang, Jianyu Li, Yi Wen, Jiping Li, Yunpeng Wang, Yongsheng Hu, Yongjie Li

**Affiliations:** ^1^Beijing Institute of Functional Neurosurgery, Xuanwu Hospital, Capital Medical University and Key Laboratory of Neurodegenerative Disease, Ministry of Education (Capital Medical University), Beijing, China; ^2^Center for Parkinson’s Disease, Beijing Institute for Brain Disorders, Beijing, China; ^3^National Institute of Neurological Disorders and Stroke, National Institutes of Health, Bethesda, MD, United States

**Keywords:** Parkinson’s disease, asymmetry, subthalamic nucleus, oscillation, firing rate, microelectrode recording

## Abstract

**Background:**

It is well known that motor features of Parkinson’s disease (PD) commonly begin on one side of the body and extend to the other side with disease progression. The onset side generally remains more severely affected over the course of the disease. However, the pathophysiology underlying the asymmetry of motor manifestations remains unclear. The purpose of the present study is **t**o examine whether alterations in neuronal activity in the subthalamic nucleus (STN) associate with PD severity.

**Methods:**

Microelectrode recording was performed in the STN during targeting for 30 patients in the treatment of deep brain stimulation. The mean spontaneous firing rate (MSFR), power density spectral analysis, and correlations were calculated. Characteristics of subthalamic oscillatory activity were compared between two hemispheres. UPDRS III scores during “Off” and “On” states were obtained for the body side of initial symptoms (BSIS) and the body side of extended symptoms (BSES).

**Results:**

There were significant differences of MSFR (41.3 ± 11.0 Hz *vs* 35.2 ± 10.0 Hz) and percentage of ß frequency oscillatory neurons (51.3% *vs* 34.9%) between BSIS and BSES. The percentage of ß frequency oscillatory neurons correlated with the bradykinesia/rigidity scores for both sides (*p* < 0.05). In contrast, the percentage of tremor frequency oscillatory neurons was significantly higher in the BSES than that in the BSIS. In particular, these neurons only correlated with the tremor scores of the BSES (*p* < 0.05).

**Conclusion:**

The results suggest that increased neuronal firing rate and ß frequency oscillatory neurons in the STN are associated with contralateral side motor severity and its progression. Tremor frequency oscillatory neurons are less observed in the STN of the BSIS suggesting that ß oscillatory activity dominates and tremor frequency oscillatory activity reciprocally declines.

## Introduction

Parkinson’s disease (PD) is a progressive neurodegenerative disorder characterized by bradykinesia/akinesia, rigidity, resting tremor, and gait instability (Marsden, 1994). The pathological hallmark of the disease is the degeneration of dopamine neurons in the substantia nigra pars compacta (Parent and Parent, 2010). The motor signs commonly begin on one side of the body and extend to the other side with disease progression. The onset side generally remains more severely affected over the course of the disease (Toth et al., 2004). These phenomena are accompanied by greater neuronal loss in the substantia nigra and more impaired putaminal uptake of ^18^F-fluorodopa contralateral to the side of initial onset (Kempster et al., 1989) or more severely affected side (Leenders et al., 1990). In particular, the clinical decline parallels the course of neuronal degeneration and an inverse linear relation between the UPDRS score and the number of nigral dopaminergic cell was observed (Lee et al., 1994; Tatsch et al., 1997). Thus, it is most likely that the asymmetry in clinical manifestations between the two sides of the body is associated with the asymmetry in the pathological progression in the two hemispheres. However, the mechanisms underlying the motor symptoms severity and its progress are still poorly understood.

The subthalamic nucleus (STN) is a favorite target for deep brain stimulation (DBS) for PD because of its fundamental role in modulating the activity of the basal ganglia (DeLong and Wichmann, 2015). It is believed that the nucleus is a main relay station of the indirect pathway of the basal ganglia, is under the control of the external pallidal segment (GPe), and directly links the cortex and cerebellum. Its action, together with signals from the direct inhibitory GABAergic pathway, modulates the activity of the globus pallidus internus (GPi) and the substantia nigra pars reticularis (SNr). Successful DBS of the STN for PD motor symptoms further implies its central role in the control of movement (DeLong and Wichmann, 2015). The implantation of DBS electrode into the STN allows microelectrode recording from this structure in both hemispheres.

Based on the classical model of pathophysiology of PD, alterations in neuronal activity in the basal ganglia are predicted to underlie parkinsonian motor symptoms (DeLong, 1990; Galvan et al., 2015). These altered neuronal activities include changes in firing rate and patterns, pathologic oscillation and synchronization in the STN and GPi. In support of the prediction, electrophysiology studies in 1-methyl-4-phenyl-1,2,3,6- tetrahydropyridine (MPTP)-treated non-human primates and in patients with PD have found an increase in firing rate, bursting activity and oscillatory activity as well as synchronization in the subthalamic neurons (Bergman et al., 1994; Hassani et al., 1996; Wichmann and Soares, 2006; Breit et al., 2007). Both microelectrode and local field potential recordings show that there are tremor frequency (4–6 Hz) oscillatory activity and β frequency (10–30 Hz) oscillatory activity observed in the STN. Close relationships between the β oscillations and rigidity–bradykinesia (Weinberger et al., 2006, 2009) and between the tremor frequency oscillations and limb tremor in the STN have been reported (Hutchison et al., 1997; Zaidel et al., 2010; Guo et al., 2013). In accordance with the model, intervention by lesioning or by high-frequency stimulation in the STN in animal model and patients with PD results in the improvement of parkinsonian symptoms (DeLong and Wichmann, 2015).

A recent study (Remple et al., 2011) demonstrated the recordings of neuronal activity from the STN of patients with early-stage PD compared with that from age- and sex-matched patients with advanced PD. They found that STN neurons have a significantly higher firing rate in advanced *versus* early-stage PD and the overall activity of the STN is significantly lower in early *versus* late PD. The data indicate that neuronal firing in the STN increases with PD progression. However, there are no significant differences between groups in the bursting or oscillatory activity in the STN.

Furthermore, some other studies found that a phase coherence in the β frequency oscillatory activity is associated with the severity of rigidity–bradykinesia (Pogosyan et al., 2010) and the spectral power at β frequency and percentage of single units oscillating at β frequency correlate with rigidity scores (Sharott et al., 2014). In particular, subthalamic neuronal oscillations and phase amplitude coupling are greater in the more affected hemisphere than that in the less affected hemisphere (Shreve et al., 2017). Although the clinical importance of the STN in PD is clear, whether the subthalamic oscillatory neurons, particular β frequency oscillatory neurons, play a critical role in PD severity and progression is still unclear.

Here, we recorded neuronal activity in the STN during targeting for the placement of DBS electrodes in PD patients. The STNs of the hemispheres contralateral to the body side of initial symptoms and contralateral to the body side of extended symptoms within individual PD patients were compared. We expect to provide important data to understand the pathophysiology underlying PD symptoms severity and its progression.

## Materials and methods

### Patients

Thirty consecutive patients (19 males and 11 females) who received bilateral DBS of the STN for PD were studied. Their mean age was 58.7 ± 7.8 years and the average disease duration was 10.2 ± 5.4 years. Diagnosis was based on the history and clinical features according to the Movement Disorder Society Clinical Diagnostic Criteria for Parkinson’s disease. MRI examination was used to exclude other neurological diseases. A pre-operative clinical assessment by a specialist was carried out using the Unified Parkinson’s Disease Rating Scale III (motor part) score during the medication “On” and “Off” states. During the Off state at the time of surgery, their mean UPDRS III score was 52.0 ± 12.5 and the Hoehn and Yahr score (Hoehn and Yahr, 1967) was 3.2 ± 0.8. Their L-DOPA equivalent daily dose was 748.2 ± 367.9 mg and all had a good response to L-DOPA with an improvement rate of 52.3 ± 10.5%. Their demographic details and clinical features are listed in Table 1.

**TABLE 1:** Demographics and characteristics of patients.

Patient	Age (years)/ gender	Handed- ness	Initially affected side	Hoehn & Yahr (Off /On)	Duration of symptoms (years)	LEDD (mg)	Response to L-dopa (%improve ment)	Lateralized UPDRS III (Off/On)		UPDRS III sub-scores (Off/On)						Oscillatory neuron type (No.) (BSIS/BSES)		
										Tremor		Rigidity		Bradykinesia				
					BSIS/BSES			BSIS	BSES	BSIS	BSES	BSIS	BSES	BSIS	BSES	Tremor frequency	β frequency	Non-oscillatory
1	65/F	L	L	2/1	5/2	800	52.0	11/5	5/0	4/2	3/0	2/0	0/0	5/3	2/0	1/2	6/4	5/4
2	58/F	R	L	2.5/2	8/2	898	58.8	13/6	10/4	0/0	0/0	5/2	4/2	8/4	6/2	3/1	4/3	2/3
3	39/M	R	L	3/2	10/5	950	65.2	22/10	15/4	9/4	6/1	4/2	4/2	9/4	5/1	3/7	4/1	3/2
4	47/M	R	L	2/1	8/2	300	65.9	22/9	11/2	7/0	1/0	3/2	2/0	12/7	8/2	2/2	2/4	7/5
5	64/F	R	L	5/3	25/20	750	49.2	17/7	17/7	0/0	0/0	6/2	6/2	11/5	11/5	2/3	10/6	1/2
6	55/F	R	R	4/2	6/3	450	57.6	27/12	16/7	5/0	0/0	7/4	5/2	15/8	11/5	0/1	3/3	5/2
7	68/M	R	R	4/3	7/5	625	36.5	24/14	18/10	5/2	1/0	7/5	7/4	12/7	10/6	1/2	10/6	0/3
8	62/M	L	L	2.5/2	6/4	1875	52.9	22/9	14/7	6/3	3/2	6/2	4/2	10/4	7/3	8/4	4/2	0/5
9	61/M	R	L	4/2	7/2	882	63.8	25/8	21/9	8/0	4/1	7/4	7/4	10/4	10/4	2/3	2/1	6/4
10	71/M	R	R	4/2	14/10	600	51.5	25/14	21/12	4/1	2/0	7/4	7/4	14/9	12/8	1/6	3/3	5/2
11	58/M	R	L	2/2	14/8	1300	65.7	13/4	12/3	2/2	2/1	6/2	6/2	5/0	4/0	3/6	4/0	2/1
12	55/M	R	R	3/2	6/1	700	57.4	20/10	16/8	1/1	1/1	7/4	5/2	12/5	10/5	0/0	2/7	2/3
13	63/M	R	R	4/2	21/16	1272	53.9	18/10	17/6	0/0	2/0	6/3	5/2	12/7	10/4	3/3	7/1	0/0
14	66/M	R	L	3/2.5	5/4	399	43.1	24/11	23/15	6/3	7/4	7/2	5/4	11/6	11/7	1/5	5/2	2/4
15	55/F	R	L	4/2	6/4	900	75.7	24/6	22/6	4/0	3/0	7/2	6/2	13/4	13/4	5/3	6/5	0/0
16	69/F	L	L	3/2.5	14/3	700	47.4	24/14	15/7	7/3	4/1	6/4	4/2	11/7	7/4	1/5	6/4	5/1
17	54/M	L	R	3/2	4/0.3	300	54.6	14/7	16/7	0/0	4/1	5/2	5/2	9/5	7/4	0/4	7/1	5/3
18	34/M	R	L	3/2	6/2	700	46.7	19/12	10/3	0/0	0/0	7/3	3/0	12/9	7/3	2/2	2/1	3/4
19	56/M	R	L	2/1.5	6/0.2	375	54.6	18/8	5/0	3/0	1/0	6/3	1/0	9/5	3/0	1/3	3/3	2/1
20	63/F	R	L	4/3	16/2	525	44.0	26/17	24/14	7/2	7/2	6/4	5/3	13/11	12/9	0/0	11/7	0/2
21	63/F	R	L	2/1.5	8/0.5	525	59.4	13/4	7/1	3/1	1/0	4/1	3/1	6/2	3/0	0/2	5/4	2/3
22	50/M	R	R	4/3	15/10	1450	38.5	25/16	19/14	3/1	0/0	8/6	7/5	14/9	12/9	1/5	8/2	1/1
23	62/M	R	L	2.5/2	20/5	800	44.7	21/9	14/8	8/1	5/0	4/2	3/2	9/6	6/6	3/5	0/1	3/2
24	62/M	R	L	4/3	15/13	400	47.5	22/12	15/6	5/2	4/1	6/3	4/2	11/7	7/3	0/4	2/1	3/1
25	58/F	L	L	3/3	6/3	350	30.0	23/15	16/10	7/7	4/4	6/3	5/2	10/5	7/4	3/1	6/1	0/3
26	61/F	R	L	3/2	10/6	1164	53.6	23/8	17/10	8/1	4/1	6/2	6/4	9/5	7/5	5/4	2/0	0/0
27	72/M	R	R	3/3	9/0.5	500	30.8	22/15	13/10	7/4	2/1	6/4	4/3	9/7	7/6	1/0	5/5	2/1
28	62/F	R	L	2.5/2	12/1	807	48.1	23/12	17/8	7/2	4/1	5/2	4/2	11/8	9/5	1/1	0/5	4/2
29	46/M	R	R	4/2	10/6	600	61.4	23/8	18/7	3/1	2/2	6/4	5/2	14/3	11/3	2/0	9/0	2/6
30	67/M	R	R	3/3	6/3	550	59.1	20/6	11/3	2/0	2/0	7/3	3/1	11/3	6/2	3/0	1/1	2/3

M, male; F, female; L, Left; R, Right; BSIS, body side of initial symptoms; BSES, body side of extended symptoms; UPDRS III, Unified Parkinson’s Disease Rating Scale Motor Score; Lateralized UPDRS III, sum of scores of items 20–26 of the UPDRS III for one side; tremor sub-score, items 20 and 21; rigidity sub-score, item 22; bradykinesia sub-score, items 23–26; LEDD: levodopa equivalent daily dosage [a dose of 100 mg levodopa are regarded as equivalent to 125 mg Levodopa and Benserazide = 133 mg Controlled-release levodopa = 100 mg Piribedil = 100 mg Amantadine = 1 mg Pramipexole = 10 mg Selegiline = 1 mg Rasagiline; when treated with Entacapone, the Levodopa dose was multiplied by 0.33 and added to the levodopa dose]. Response to L-dopa (% improvement) = UPDRS III [(“OFF” score-“ON” score)/“OFF” score × 100%].

The lateralized UPDRS sub-score (Cubo et al., 2010) was calculated as the sum of the tremor score (items 20 and 21), rigidity (item 22), and bradykinesia (items 23–26) scores of one side of the body (total possible score 36). The body side of initial symptoms (BSIS) group and the body side of extended symptoms (BSES) groups were determined based on: (1) each patient’s own report of the side on which the first symptom started and on which the symptom extended; (2) the duration of symptoms on each side; (3) the lateralized UPDRS III Off medication sub-score. The working definition of BSIS and BSES is in connection with the recording sides of the contralateral hemispheres.

Table 2 lists the detailed information of the two groups. If the onset symptom was axial, the first lateralized symptom was used.

**TABLE 2:** Duration of symptoms and UPDRS III sub-scores in the BSIS and BSES.

Patients	*n* = 30		
Age (years)	58.9 ± 8.8		
Gender (female/male)	11/19		
Handedness (right/left)	25/5		
Hoehn &Yahr (off/on)	3.2 ± 0.8/2.2 ± 0.6		
UPDRS III total score (off/on)	52.0 ± 12.5/25.0 ± 8.9		
**Items**	**BSIS**	**BSES**	**Differences between the BSIS and BSES**
Duration of symptoms (years)	10.2 ± 5.3	4.8 ± 4.8	5.4 ± 3.4****
Body side of motor symptoms (left/right)	20/10	10/20	10/10
Lateralized UPDRS III sub-scores (off/on)	20.8 ± 4.3/9.9 ± 3.6	15.2 ± 4.8/6.9 ± 4.0	5.6 ± 3.6***/3.0 ± 3.0***
Tremor score (items 20 and 21) (off/on)	4.4 ± 2.9/1.4 ± 1.6	2.6 ± 2.0/0.8 ± 1.1	1.7 ± 2.2***/0.6 ± 1.2**
Rigidity score (item 22) (off/on)	5.8 ± 1.3/2.9 ± 1.3	4.5 ± 1.7/2.2 ± 1.3	1.3 ± 1.3***/0.7 ± 1.2**
Bradykinesia score (items 23–26) (off/on)	10.6 ± 2.5/5.6 ± 2.4	8.0 ± 3.0/4.0 ± 2.5	2.5 ± 1.5***/1.7 ± 1.8***

BSIS, body side of initial symptoms; BSES, body side of extended symptoms; UPDRS III, Unified Parkinson’s Disease Rating Scale Motor Score; Lateralized UPDRS III, sum of scores of items 20–26 of the UPDRS III measured on one side. Values are expressed as the mean ± SD; **p < 0.01, ***p < 0.001, ****p < 0.0001.

The research was conducted based on the Declaration of Helsinki and was approved by the Xuanwu Hospital Ethics Committee, Capital Medical University. Patients gave written informed consent.

### Surgical procedure and electrophysiology

The detailed methods are described in previous studies (Hutchison et al., 1997; Guo et al., 2013). A sagittal magnetic resonance image (Siemens 1.5 Tesla, Sonata, Germany) using the Cosman-Roberts-Wells frame (Radionics, Burlington, MA, USA) was obtained prior to the stereotactic surgery and then, fused with a CT scan using Framelink software (Medtronic StealthStation S7, Medtronic Navigation Inc., Louisville, KY, USA). The STN was calculated based on a human atlas. The coordinates of the target of the STN were: 12–13 mm lateral, 2–3 mm posterior, and 4–6 mm inferior to the midcommissural point. The anteroposterior and lateromedial angles were about 60°and 12°, respectively.

Microelectrode recording (the electrode at tip 10–20 μm with impedance 0.4–1 MΩ) was performed to explore the border and length of the STN. Once the electrode entered the STN, there was immediately increased background noise with typically multiple discharges with high frequency and bursting activity. When the electrodes reached the ventral border of the STN, the background noise gradually decreased until the substantia nigra pars reticulata was reached. There was a high frequency discharge with more regular firing and lower amplitude discharges compared with subthalamic neurons (Du et al., 2018).

We usually recorded all isolated units for between 15 s and several minutes to explore the spontaneous firing rates, patterns and neuronal oscillatory activity.

Recorded signals from the microelectrode were amplified (× 20,000), filtered (100 Hz–20 kHz bandpass), and sampled at 12 kHz. All recordings were obtained using the MicroGuide system (AlphaOmega Engineering, Nazareth, Israel) with patients at rest.

Pre-operation, patients were requested to withdrawn their medications at least 12 h before surgery. They were awake during the entire operation to ensure cooperation.

### Data analysis

All the microelectrode recordings and EMG signals were transformed into the Spike 2 software (Cambridge Electronic Design, Cambridge, UK) for further analysis. Only stable, well-isolated single neurons observed in recordings longer than 15 s without artifacts were selected to further analysis. Spikes with a signal-to-noise ratio > 2:1 were used. The interspike interval (ISI), the ISI histogram, and the coefficient of variation of the ISI (CV) were calculated to study the mean spontaneous firing rate (MSFR) and patterns. Power spectral density (PSD) analysis (A Hanning window at 50% overlap between two windows) was used to identify neuronal oscillation. The significant oscillatory frequencies were defined when exceeding a threshold of 5 SD above the mean power in the 10 –100 Hz band (Moran et al., 2008). Neurons with three oscillatory patterns were classified: (1) tremor frequency oscillatory neuron (the frequency of peak power at range of 3–6 Hz); (2) β frequency oscillatory neuron (the frequency of peak power at range of 8–35 Hz); and (3) non-oscillatory neurons (neuron without any peak power). The proportion of different patterns of oscillatory neuron was calculated based on the number patterns of oscillatory neurons divided by the total number of neurons identified. Correlation and linear regression analysis was carried out to study the relationship between pattern of oscillatory neurons and UPDRS sub-scores (Shreve et al., 2017). All data analysis was performed using Spike II 7.02 (Cambridge Electronic Design, Cambridge, UK), GraphPad Prism 7 (GraphPad Software Inc., San Diego, CA, USA), and Origin 7.5 (OriginLab Corp., Northampton, MA, USA).

### Statistical analysis

All data are illustrated as the mean ± standard deviation (SD). The means of two normally-distributed variables were compared using independent samples Student’s *t*-test. The Mann-Whitney test was used to compare the means of two groups of variables not normally distributed. The neuronal firing rates of the three patterns of neuron within and between groups was using two-way analysis of variance (ANOVA) and the Bonferroni multiple comparison *post hoc* test, with a level of significance of α = 0.05. The different patterns of oscillatory neurons in the groups were compared using χ^2^ analysis and Fisher’s exact test. Linear regression and Pearson correlation analysis was used to assess the correlation of oscillatory neurons with UPDRS sub-scores. Statistical significance was set at *p* < 0.05.

SPSS 17.0 (SPSS, Chicago, IL, USA), GraphPad Prism 7 (GraphPad Software Inc., San Diego, CA, USA), and Origin 7.5 (OriginLab Corp., Northampton, MA, USA) were used for statistical analysis.

## Results

Five hundred seventy-one neurons were identified in 60 STNs of 30 patients, 30 STNs associated with the BSIS and 30 STNs associated with the BSES. The mean duration of the neuronal recordings (17.6 s ± 6.3 s *vs* 16.5 s ± 4.6 s) and the length of the STN (5.1 ± 1.0 mm *vs* 5.0. ± 1.0 mm, all *p* > 0.05, paired *t*-test) showed no significant difference between the BSIS and BSES. The main analysis focused on oscillatory neurons.

### Clinical characteristics of the BSIS and BSES groups

Significant differences in the mean duration of symptoms (10.2 ± 5.3 vs 4.8 ± 4.8 years), lateralized UPDRS III (20.8 ± 4.3 *vs* 15.2 ± 4.8), tremor sub-score (4.4 ± 2.9 *vs* 2.6 ± 2.0), rigidity sub-score (5.8 ± 1.3 *vs* 4.5 ± 1.7), and bradykinesia sub-score (10.6 ± 2.5 *vs* 8.0 ± 3.0) were found between the BSIS and BSES groups in the “Off” medication condition (all *p* < 0.05, paired *t*-test) as well as in the “On” state.

### Analysis of spontaneous firing rates and proportion of oscillatory and non- oscillatory neurons in the STNs of BSIS and BSES

Of all the recorded neurons, 271 were from the STNs of the BSIS (*n* = 30 patients). PSD analysis showed that 51.3% (139/271) were ß-frequency neurons with mean peak power at 18.6 ± 7.3 Hz (median, 16.9 Hz; range, 8–35 Hz) and 21.4% (58/271) were tremor-frequency neurons with mean peak power at 4.8 ± 1.1 Hz (median, 4.8 Hz; range, 3–7 Hz). The remaining 27.3% (74/271) were non-oscillatory neurons.

ISI analysis showed that the MSFR of the 271 neurons was 41.3 ± 11.0 Hz (median, 40.8 Hz; range, 13.6–85.7 Hz). Further analysis showed that the MSFR of ß-frequency neurons was 42.1 ± 11.4 Hz (median, 41.4 Hz; range, 16.3–85.7 Hz), that of tremor-frequency neurons was 39.5 ± 11.4 Hz (median, 40.8 Hz; range, 13.6–72.9 Hz), and of non-oscillatory neurons was 41.1 ± 10.0 Hz (median, 39.8 Hz; range, 22.0–65.4 Hz).

Of the 241 neurons from the STN of the BSES (*n* = 30), PSD analysis showed that 34.9% (84/241) were ß-frequency neurons with mean peak power at 19.8 ± 7.6 Hz (median, 19.8 Hz; range, 8.0–35.0 Hz) and 34.9% (84/241) were tremor-frequency neurons with mean peak power at 4.5 ± 1.1 Hz (median, 4.4 Hz; range, 3.0–7.3 Hz). The remaining 30.2% (73/241) were non-oscillatory neurons.

ISI analysis showed that the MSFR of all 241 neurons was 35.1 ± 10.0 Hz (median, 35.3 Hz; range, 14.7–94.3 Hz). Further ISI analysis showed that the MSFR of ß-frequency neurons was 36.3 ± 10.2 Hz (median, 36.2 Hz; range, 16.1–94.3 Hz), that of the tremor-frequency neurons was 34.7 ± 9.9 Hz (median, 35.3 Hz; range, 15.7–60.8 Hz), and of non-oscillatory neurons was 34.4 ± 10.1 Hz (median, 35.1 Hz; range, 14.7–66.4 Hz).

**FIGURE 1:**
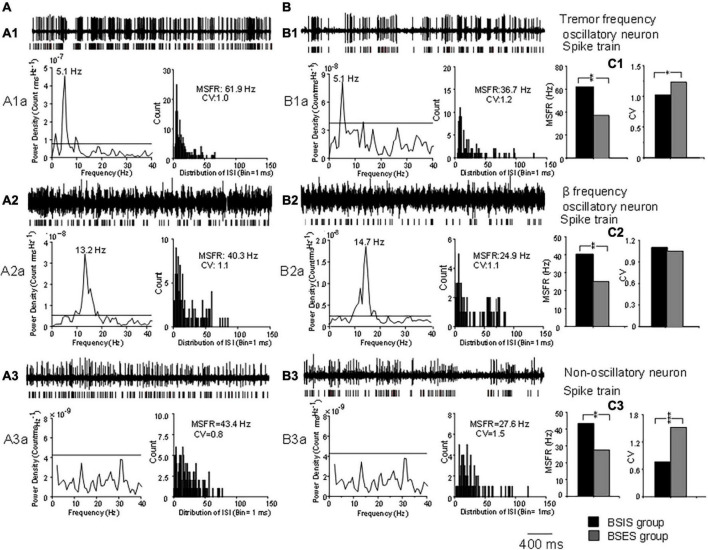
Characteristics of oscillatory and non-oscillatory neurons in the STN in BSIS and BSES groups. **(A,B)** Representative recordings from single oscillatory and non-oscillatory neurons in the STN in the BSIS and BSES. **(A1,B1)** Patterns of tremor-frequency neurons. **(A2,B2)** β-frequency neurons. **(A3,B3)** Non-oscillatory neurons. Upper traces, raw data; lower traces, spike trains. A1a-A3a and B1a-B3a show the corresponding power spectral analysis and ISI histogram with the MSFR and CV. **(C1–C3)** MSFRs and CVs of the three types of neurons in the BSIS and BSES. Horizontal line indicates the significant oscillatory level; ISI, inter-spike interval; MSFR, mean spontaneous firing rate; CV, coefficient of variation of ISI; BSIS, body side of initial symptoms; BSES, body side of extended symptoms; *p < 0.05, **p < 0.01.

Representative examples of tremor-frequency, β-frequency, and non-oscillatory neurons recorded from the STN in the BSIS and BSES and their firing rates and CVs are shown in Figure 1.

### Comparisons of MSFR and proportion of subthalamic oscillatory and non-oscillatory neurons between BSIS and BSES

**FIGURE 2:**
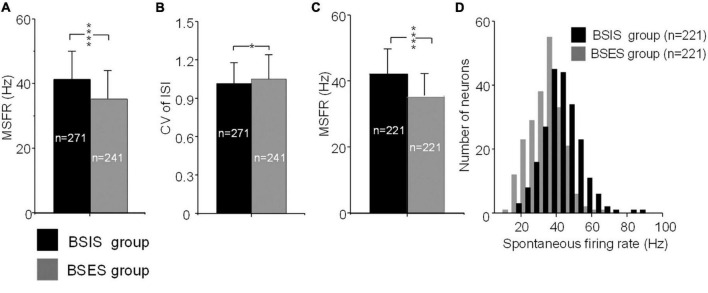
MSFRs and CVs of STN neurons in BSIS and BSES groups. **(A)** MSFRs of STN neurons in the BSIS are significantly higher than in the BSES (****p < 0.0001). **(B)** CVs of STN neurons in the BSIS are significantly smaller than in the BSES (*p < 0.05). **(C)** MSFRs of STN neurons in the BSIS group are significantly higher than in the BSES group (matched numbers of neurons in the two groups). **(D)** The distribution of spontaneous STN firing rates is significantly higher in the BSIS than in the BSES (bin size, 5 Hz). MSFR, mean spontaneous firing rate; CV, coefficient of variation of ISI; ISI, inter-spike interval; STN, subthalamic nucleus; BSIS, body side of initial symptoms; BSES, body side of extended symptoms.

The MSFR of subthalamic neurons recorded from the BSIS was significantly higher than that of the BSES (41.3 ± 11.0 *vs* 35.1 ± 10.0 Hz) and this difference was statistically significant (*t* = 6.50, *p* < 0.0001; Figure 2A). In accordance with the MSFR, the CV of ISIs in the BSIS was smaller than that in the BSES (1.02 ± 0.16 *vs* 1.05 ± 0.19, *t* = –2.26, *p* < 0.024; Figure 2B). We further compared the spontaneous firing rates of subthalamic neurons on each side (*n* = 221) in the BSIS and BSES. Again, a significant difference in the MSFR was found between sides (41.9 ± 10.8 Hz *vs* 35.0 ± 9.4 Hz, *p* < 0.0001, paired *t*-test, Figure 2C). Histogram showing distribution of spontaneous firing rate revealed that neuronal firing rates in the STN of the BSIS were significantly higher than in the BSES (Figure 2D).

**FIGURE 3:**
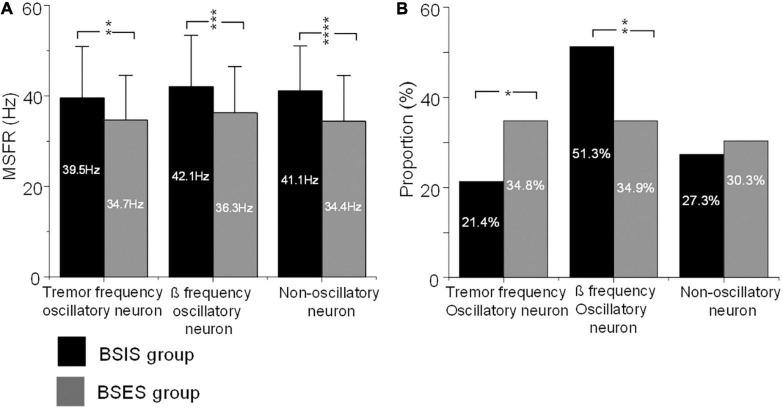
MSFRs and proportions of the three types of oscillatory and non-oscillatory neuron in BSIS and BSES groups. **(A)** MSFRs of tremor-frequency, β-frequency, and non-oscillatory neurons in the BSIS group are significantly higher than in the BSES group (**p < 0.01, ***p < 0.001, ****p < 0.0001). **(B)** The proportion of β-frequency neurons is significantly higher while that of tremor-frequency neurons is significantly lower in the BSIS group than in the BSES group (*p < 0.05). There is no significant difference in the proportion of non-oscillatory neurons between groups (*p* = 0.67). MSFR, mean spontaneous firing rate; BSIS, body side of initial symptoms; BSES, body side of extended symptoms.

Further ANOVA indicated significant differences of the MSFR of the three patterns of subthalamic neurons between the two sides (*F* = 35.4, df = 1, *p* < 0.0001). Bonferroni tests showed that there were no significant differences of the MSFR of three patterns of neurons within the sides (all *p* > 0.05), but there were significant differences of the MSFR for all patterns of neurons between the BSIS and BSES (all *p* < 0.05). Comparison of the BSIS and BSES revealed that the MSFR of tremor-frequency neurons was 39.5 ± 11.4 vs 34.7 ± 9.9 Hz, of β-frequency neurons was 42.1 ± 11.4 Hz *vs* 36.3 ± 10.2 Hz, and of non-oscillatory neurons was 41.1 ± 10.0 *vs* 34.4 ± 10.1 Hz (all *p* < 0.01; Figure 3A). The results further showed that the MSFR in the STN of the BSIS was higher than that in the BSES.

The χ^2^ test showed significant differences in the proportions of patterns of subthalamic oscillatory neurons between the BSIS and BSES (*p* < 0.001). Further comparisons showed that the proportion of β frequency oscillatory neurons in the BSIS was significant higher than that in the BSES group (51.3 *vs* 34.9%, *p* < 0.001). In contrast, the proportion of tremor frequency oscillatory neurons in the BSES was higher than those in the BSIS (34.9 *vs* 21.4%, *p* < 0.001). There was no significant difference in the proportion of non-oscillatory neurons between the two sides (*p* = 0.49; Figure 3B).

### Correlation of subthalamic oscillatory neurons with pre-operative UPDRS sub-scores in the BSIS and BSES

**FIGURE 4:**
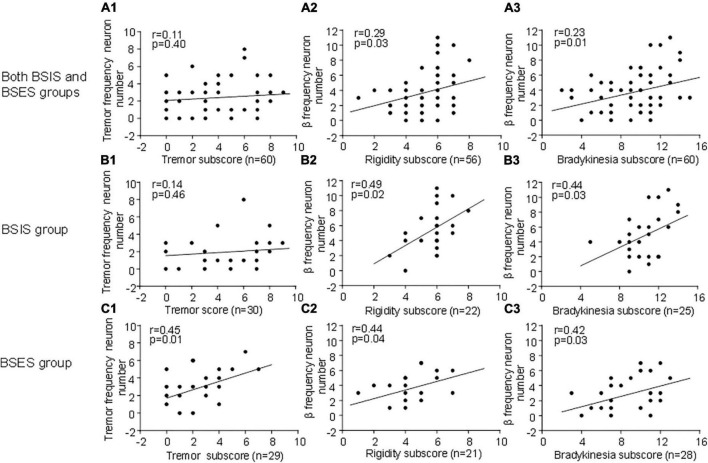
Correlation of oscillatory neurons with UPDRS III sub-scores across groups during the “Off” medication state. **(A1,B2)** STN tremor-frequency neurons are not correlated with the UPDRS III tremor sub-score in the BSIS (*p* > 0.4 0.46), while they are significantly correlated with the tremor sub-score in the BSES (*p* < 0.01). **(A2–C3)** STN β-frequency neurons are significantly correlated with the UPDRS III rigidity and bradykinesia sub-scores across all groups during the “Off” state; the relationship is linear (*p* < 0.01–0.04).

To determine whether subthalamic oscillatory neurons are associated with parkinsonian symptoms, we correlated the tremor-frequency and β frequency oscillatory neurons with the UPDRS III sub-scores for tremor, bradykinesia, and rigidity. We found that overall, the β frequency oscillatory neurons were significantly correlated with the UPDRS III rigidity and bradykinesia sub-scores during the “Off” state across both sides, showing a linear relationship (Figures 4A2–C3). The results suggest that β-frequency neurons are closely associated with rigidity and bradykinesia (r, 0.23–0.44, *p* = 0.01–0.04). Conversely, tremor-frequency neurons were only significantly correlated with the tremor sub-score in the BSES (r, 0.4, *p* < 0.01), but not with that in the BSIS (r, 0.11–0.14; *p* = 0.40–0.46; Figures 4A1–C1).

## Discussion

Here, we compared for the first time the subthalamic oscillatory activity in the hemispheres between BSIS and BSES in individual PD patients. There were three findings. First, the MSFR of neurons in the STN of the BSIS was significantly higher than that of the BSES. Second, the proportion of β frequency oscillatory neurons was higher in the BSIS than that in the BSES. Overall, the correlation of β frequency oscillatory neurons with UPDRS sub-scores of rigidity and bradykinesia was greater in the BSIS than that in the BSES. Third, the tremor frequency oscillatory neurons were fewer in the BSIS than in the BSES. The results indicated that increased firing rate and ß frequency oscillatory neurons in the STN were more frequent on the hemisphere opposite the more severe bradykinesia. These findings support the view that increased neuronal firing and ß frequency oscillatory neurons in the STN are associated with severity of PD and its progression. Conversely, tremor frequency oscillatory neurons are less frequent in the STN of the BSIS suggesting that ß oscillatory activity dominates and tremor frequency oscillatory activity reciprocally declines.

Our finding of a17.7% increased STN firing rate in the BSIS versus the BSES (41.3 ± 11.0 Hz vs. 35.1 ± 10.0 Hz) of the individual patients is consistent with the recent report (Remple et al., 2011) which found a 26.4% elevation in the STN firing rate of advanced stage *versus* early stage groups (36.3 *vs.* 28.7 Hz). These data support the prediction that STN neuronal firing rate increase with PD severity and its progression. According to pathophysiology “rate model” of parkinsonism, the deficits of dopaminergic input to the striatum result in disinhibition of the STN, causing overdrive the output nuclei of GPi and SNr, leading to increase inhibition of the motor thalamus, resulting in the cardinal symptoms of PD, such as akinesia and bradykinesia (DeLong, 1990). Our findings with the results of Remple et al. (2011) are consistent with the report from studies of animal model that show increased discharge rates in the STN following the application of toxic compounds to the substantia nigra (Bergman et al., 1994; Hassani et al., 1996; Wichmann and Soares, 2006; Breit et al., 2007). Examples show that monkeys exhibit a 36.8% increase in mean firing rate (19 *vs.* 26 Hz) when given a dose of MPTP sufficient to create severe parkinsonian symptom(Bergman et al., 1994); an increase in the average discharge rate of STN neurons from a control value of 25.7 Hz to 36.1 Hz has been reported in parkinsonian monkeys (DeLong, 1990). Accordantly, the firing rate of subthalamic neurons seems to be increased at an early stage of the disease, when patients have minor or mild symptoms, and becomes more elevated as the disease progresses and symptoms worsen. In our case, a finding was that patients had significant difference in the duration of symptoms between the BSIS and BSES groups. In accordance with the symptom duration, there was a significant difference in the lateralized UPDRS III scores between the two groups (20.8 ± 4.3 *vs* 15.2 ± 4.3, *p* < 0.01) during the “Off” state (Table 2). Thus, the increased MSFR in the STN of the BSIS group was most likely associated with more severe symptoms. Our findings, along with those of others (Remple et al., 2011) strongly support the rate model of PD pathophysiology (Albin et al., 1989; DeLong, 1990).

In this study, we demonstrated that the proportion of β frequency oscillatory neurons was higher in the STN contralateral to the BSIS than that in the STN contralateral to the BSES. Furthermore, correlations of β frequency oscillatory neurons with the UPDRS sub-scores for rigidity and bradykinesia in the BSIS were greater than those in the BSES. The results suggest that ß frequency oscillatory neurons in the STN are more frequent on the hemisphere opposite the more severe bradykinesia and rigidity. The findings are also consistent with a recent study which demonstrated that subthalamic neurons with β frequency oscillations and phase amplitude coupling are greater in the more affected hemisphere in PD (Shreve et al., 2017).

A large number of physiological studies (Brown et al., 2001; Cassidy et al., 2002; Levy et al., 2002; Priori et al., 2002; Kuhn et al., 2004, 2008, 2009; Doyle et al., 2005) have suggested that basal ganglia β frequency oscillatory neurons in the 8–35 Hz range are involved in parkinsonian rigidity–bradykinesia (Brown, 2003). Single units and local field potentials recorded in the STN from patients with PD have demonstrated that there is a relationship between β frequency oscillatory activity and rigidity–bradykinesia (Weinberger et al., 2006, 2009). Decreased β oscillation is correlated with the degree of improvement in rigidity–bradykinesia after dopamine medication (Zaidel et al., 2010). A significant correlation between L-dopa-induced β-frequency power suppression and rigidity–bradykinesia has been reported (Pogosyan et al., 2010). The β activity in the STN is also suppressed by DBS in parallel with improved motor performance in patients with PD (Ray et al., 2008; Pogosyan et al., 2010; Zaidel et al., 2010). Furthermore, a recent study found synchrony of the β oscillation in the STN and the severity of parkinsonian rigidity–bradykinesia (Weinberger et al., 2009). Moreover, a recent study (Feng et al., 2016) showed that STN β frequency oscillatory neurons are coherent with limb muscular activity in PD patients with the akinetic/rigid type. The proportion of β frequency oscillatory neurons is higher in the STN of the akinetic/rigid type than in the mixed type. All of the above evidence indicates a close relationship between subthalamic β oscillatory neurons and rigidity/bradykinesia.

In contrast, we found that the proportion of tremor-frequency neurons in the STNs of the BSES was higher than that in the BSIS (34.9 *vs* 21.4%). A significant correlation of tremor-frequency neurons with the tremor score in the BSES was also found. The results suggest that tremor-frequency neurons are less pronounced in the hemisphere contralateral to initiate symptom with progression. Similar findings (Toth et al., 2004) have also been reported in a clinical study. In that study, 33 patients followed for 10 years who had parkinsonian signs of greatest severity on one side but subsequently over ∼ 5.4 years, the signs gradually became more prominent on the opposite side. Forty-five percent of these patients demonstrated evolution to a rigid form of parkinsonism with disappearance of rest tremor over an average of 7.1 years after presentation. The conclusion is that tremor becomes less pronounced with progression of the disease in some patients.

The pathophysiology of PD tremor certainly differs from that underlying the akinesia/bradykinesia and rigidity (Zaidel et al., 2009). The clinical manifestations are separate, and the response of tremor to L-dopa agents is lack certain than that of bradykinesia (Hallett, 2012, 2014). Tremor often occurs early in the disease. It can occur on the body side contralateral to the otherwise most affected side where bradykinesia and rigidity are most prominent. Its severity is not always correlated with that of akinesiai/bradykinesia or rigidity and does not worsen at the same rate as akinesia/bradykinesia (Jankovic and Kapadia, 2001; Zaidel et al., 2009). Moreover, unlike rigidity and bradykinesia, the tremor severity is not often associated with striatal dopamine deficits. This suggests that non-L-Dopa mechanisms likely outside the basal ganglia play an additional important role (Helmich et al., 2012). Our findings further support the idea that the pathophysiology of PD tremor differs from that of akinesia/bradykinesia and rigidity.

The clinical feature of PD is that the motor signs usually start on one body side in an asymmetrical fashion and spreads to the other side with disease progression. The onset side generally remains more severe along the course of the disease (Leenders et al., 1990). In the current study, 30 patients present a clear unilateral motor symptom onset that gradually spread to other side after a mean time of 5.4 ± 3.4 years. The significantly different lateralized UPDRS III sub-scores for bradykinesia, rigidity, and tremor between BSIS and BSES indicated that the side with initial symptoms remains more severe with disease progression. The finding was supported by postmortem pathological studies which have shown greater neuronal loss in the substantia nigra contralateral to the initially affected side (Kempster et al., 1989). Further positron emission tomography studies demonstrated that putaminal uptake of ^18^F-flurodopa is more impaired contralateral to the initially more affected side (Leenders et al., 1990). Our findings indicated that increase in firing rate and β frequency oscillatory neurons are associated with the clinically more severe body side in PD patients.

In the current study, the clinical and physiological information was collected from each side of the body within individuals, so the age, gender, and L-DOPA equivalent daily dose did not influence our results. The major limitation was the small sample size of 30 patients that might decrease statistical power. Additionally, considering the issue of STN nucleus sub-region, there may be differences in neuronal cells of different regions (motor, limbic, associative). The comparison of mean values used in this study was not corrected by neuroimaging, limiting the ability to define electrophysiological indicators based on sub-region. Another limitation was that we only collected pre-surgery UPDRS III data but without data of post- surgery, so that the clinical outcome of patients was not evaluated. In addition, previous studies have demonstrated that right-handed patients tend to have initial motor symptoms on the right side (Kempster et al., 1989; Toth et al., 2004) suggesting left hemisphere predominance of nigrostriatal dysfunction in PD. However, in the current study, 83.3% (25/30) of the patients were right-handed and 36.0% (9/25) of them had initial motor symptoms on right side. Thus, the hypothesis of right-handedness and the left hemisphere dominance of nigrostriatal dysfunction in PD cannot fully explain this phenomenon, so other factors might be involved. No doubt, more strict inclusion criteria in future studies with larger patient populations are needed to further confirm the findings.

## Conclusion

The findings suggest that the increased neuronal firing rate and proportion of ß frequency oscillatory neurons are associated with severity of PD, such as akinesia/bradykinesia, and its progression. These findings support the pathophysiology model of PD that the altered neural activities underlie parkinsonian motor symptoms. The reduced tremor frequency oscillatory neurons in the STN of the BSIS suggests that ß frequency oscillatory activity takes over the basal ganglia making it difficult for tremor neuronal activity to sustain itself. Our data also support the clinical observations that the onset of motor symptoms in PD is usually asymmetric and the side on which they are initiated is often more severely affected over the course of the disease (Toth et al., 2004).

## Data availability statement

The original contributions presented in this study are included in the article/supplementary material, further inquiries can be directed to the corresponding author.

## Ethics statement

The research was conducted based on the Declaration of Helsinki and was approved by the Xuanwu Hospital Ethics Committee, Capital Medical University. All patients signed a written informed consent for the surgery that involved microelectrode recording.

## Author contributions

XMZ and PZ contributed by collecting the data, analysis and interpretation of the data, and drafting and revising the manuscript. MH was involved in critically revising the manuscript and provided the intellectual guidance. YQZ, JYL, YW, JPL, YPW, YSH, and YJL contributed to the surgery, data collection, and conceptualization of the study. All authors contributed to the article and approved the submitted version.
